# Dynamic Tracking of Tumor Microenvironment Modulation Using Kaede Photoconvertible Transgenic Mice Unveils New Biological Properties of Viral Immunotherapy

**DOI:** 10.1158/2767-9764.CRC-24-0434

**Published:** 2025-02-17

**Authors:** Anne R. Diers, Qiuchen Guo, Zhi Li, Erin Richardson, Suaad Idris, Claire Willis, Paul P. Tak, David R. Withers, Francesca Barone

**Affiliations:** 1 Candel Therapeutics, Inc., Needham, Massachusetts.; 2 Institute of Immunology and Immunotherapy, College of Medical and Dental Sciences, University of Birmingham, Birmingham, United Kingdom.

## Abstract

CAN-2409 is a replication-defective adenovirus that delivers the herpes simplex virus–thymidine kinase gene to infected cells. Intratumoral administration of CAN-2409, followed by prodrug, results in the formation of a toxic metabolite able to induce immunogenic cell death, exposure of tumor-associated antigens, and activation of local and systemic immune responses. We used a dynamic labeling model with MC38 tumor cells implanted in photoconvertible Kaede mice. Violet light was used to label the tumor microenvironment (TME), distinguishing retained versus newly entering cells and allowing real-time monitoring of immune compartment changes within tumors. Administration of CAN-2409 + prodrug led to control of tumor growth and significantly increased effector CD8^+^ T-cell responses. Photolabeling of the TME revealed that rather than enhancing recruitment of T cells to the tumor, CAN-2409 altered the TME whereby newly entering and retained CD8^+^ T cells were significantly more proliferative. CAN-2409 supported reinvigoration of tumor-associated antigen–specific CD8^+^ T cells and expansion of regulatory T cells of an altered phenotype. Moreover, the combination of CAN-2409 + prodrug and anti–CTLA-4 antibody treatment further improved control of tumor growth, in part by the enhanced CD8^+^ T-cell–mediated effector function and diminished regulatory T cell–mediated immunosuppression. Collectively, these data defined at least two temporally distinct pathways underpinning the mechanism of action of CAN-2409 that overcome cell exhaustion and decrease immune suppression. The results also support the rationale for future clinical trials of CAN-2409 treatment combined with anti–CTLA-4 antibody therapy.

**Significance:**

This study utilized a novel photoconvertible mouse tumor model to track immune cell trafficking upon treatment with an investigational viral immunotherapy (CAN-2409), revealing enhanced T-cell responses after viral immunotherapy associated with local proliferation of T cells within tumors that could further enhance antitumor efficacy in combination with immune checkpoint inhibitors. These findings define temporally and spatially distinct interactions of immune cells that could be harnessed by novel therapeutics.

## Introduction

The generation of antitumor immunity requires cellular interactions both within the tumor and in draining lymphoid tissue. Exactly where the generation and persistence of different effector T-cell populations are regulated remains unclear, impeding precise manipulation of the response through therapeutic intervention. Virus-mediated *in situ* vaccination has emerged as a therapeutic strategy to alter the tumor microenvironment (TME) and enhance therapeutic immune-mediated antitumor responses against the injected tumor and uninjected metastases (1, 2). CAN-2409 is a replication-defective adenovirus that delivers the herpes simplex virus–thymidine kinase gene when directly injected into a tumor. Intratumoral (i.t.) administration of CAN-2409, followed by prodrug, results in local herpes simplex virus-thymidine kinase–mediated formation of a cytotoxic metabolite that induces immunogenic cell death and exposure of tumor-associated antigens (TAA) to the immune system. At the same time, the adenoviral serotype 5 capsid protein elicits a strong proinflammatory signal in the TME. Together, this creates the optimal conditions to induce an individualized and specific CD8^+^ T-cell–mediated response for broad local and systemic antitumor activity (3, 4).

Accordingly, treatment with CAN-2409 + prodrug (from now on referred as CAN-2409) in animal models of cancer not only resulted in local tumor control and increased survival but also in protection against metastases and tumor rechallenge (5–7). CAN-2409–treated tumors were characterized by infiltration of immune cells, including macrophages and T cells and increased expression of Th cell type 1 cytokines (7–10). Notably, the antitumor effect of CAN-2409 was abrogated upon depletion of CD8^+^ T cells and adoptive transfer of CD8^+^ T cells from CAN-2409–treated mice yielded antitumor immunity in naïve tumor–bearing mice (5–7), providing evidence of effective CD8^+^ T-cell–mediated vaccination against the tumor.

Clinical investigation of CAN-2409 has been undertaken in multiple solid tumor indications, including high-grade glioma (11, 12), prostate cancer (13, 14), ovarian cancer (15), retinoblastoma (16), pancreatic cancer (17), malignant pleural effusion (18), pediatric high-grade glioma (19), and non–small cell lung cancer (NSCLC; ref. 4). This therapeutic approach has generally been well tolerated with promising early efficacy signals. Biopsy or resection samples obtained from patients treated with CAN-2409 consistently showed the presence of CD8^+^ cytotoxic T cells after treatment (3, 4, 17, 20). Recently, results from an ongoing phase 2 clinical trial of CAN-2409 + prodrug in patients with NSCLC with an inadequate response to anti–PD-1/PD-L1 immune checkpoint inhibitor (ICI) therapy demonstrated infiltration by cytotoxic T cells within tumors after CAN-2409 treatment and increased proximity of the CD8^+^ T cells to cancer cells based on spatial analysis (21, 22). In the same patients, analysis of peripheral blood mononuclear cells showed increased proliferation and activation of circulating CD8^+^ T cells, as shown by increased expression of Ki67, granzyme B (GZB), and IFNγ. Consistent with the generation of circulating TAA-specific memory T cells, clinical responses have been observed in uninjected sites, strongly supporting the hypothesis that CAN-2409 + prodrug treatment represents a systemic immunotherapy, delivered locally.

The mechanisms involved in the evolution of the local immune response to CAN-2409 + prodrug in injected lesions into systemic antitumor immunity, including changes in the TME and tumor-draining lymph node (dLN), are not completely understood. Moreover, the interplay between antitumor versus antiviral immune responses activated by viral immunotherapies like CAN-2409 and the contribution of the antiviral response to antitumor efficacy remain poorly defined (23–25). Recent studies have exploited photoconvertible mice to temporally and specifically label the immune compartment of tumors and then track the recruitment and retention of immune cells (26–28). In this study, we reasoned that the use of such models would clarify how CAN-2409 + prodrug alters the antitumor T-cell response, informs where changes in the T-cell response are observed, and elucidates the mechanisms altering their composition in the tumor. To this end, tumors were implanted in Kaede photoconvertible mice, photolabeled, and tumor and dLN immune compartments were assessed. These studies revealed that the enhanced T-cell response after CAN-2409 treatment is associated with local proliferation of T cells within the tumor after trafficking to the tissue. These findings supported the evaluation of potential synergy between CAN-2409 treatment and anti–CTLA-4 antibodies, and it was found that the CAN-2409–dependent changes in the tumor immune compartment support enhanced tumor control with combination therapy.

## Materials and Methods

### Mice

Female C57/BL/6 mice were purchased from Charles River Laboratories or The Jackson Laboratory (RRID: IMSR_JAX:000664) and allowed to acclimate for at least 1 week prior to studies. C57BL/6 Kaede mice were maintained and bred at the University of Birmingham Biomedical Services Unit. Animals were used in accordance with institutional guidelines and were approved by the University of Birmingham Animal Welfare and Ethical Review Body prior to execution. During the study, the care and use of animals were conducted in accordance with the regulations of the Association for Assessment and Accreditation of Laboratory Animal Care.

### Subcutaneous tumor model and photoconversion

MC38 murine colon adenocarcinoma cells (RRID: CVCL_B288) were kindly provided by Dr. Gregory Sonnenberg (Weill Cornell Medicine) in December 2017. Cells were cultured in RPMI supplemented with 2 mmol/L L-glutamine (21875034; Thermo Fisher Scientific), 10% FBS (F9665; Sigma-Aldrich), and penicillin–streptomycin (P4333; Sigma-Aldrich) at 37°C and with 5% CO_2_ and tested periodically for *Mycoplasma* using either PlasmoTest or MycoStrip kits from Invitrogen. After thawing and passaging twice over 5 days, log-phase cells were harvested and suspended in serum-free media or Dulbecco’s PBS, and 100 µL of cell suspension containing 2.5 × 10^5^ (in Kaede hosts) or 1 × 10^6^ cells was subcutaneously injected into mice [wild-type (WT) or Kaede C57BL/6] in the preshaved rear flank area under anesthesia. Tumor size was periodically measured using Vernier calipers, and the volume was expressed in cubic millimeters. Tumor weight was measured at the endpoint of the experiment. Tumors were photoconverted by exposure to a 405-nm wavelength LED light from a fixed distance of 1 cm using a Dymax BlueWave QX4 system (DYM41572; Intertronics), outfitted with an 8-mm focusing lens for a total of 3 minutes at 50% of full power with a 5-second break for every 20 seconds, as described previously (28). Surrounding areas were shielded using a black cardboard.

### Treatment

Mice bearing palpable tumors were randomized, and then vehicle control or CAN-2409 was administered intratumorally (20 μL of 1.7 × 10^11^ viral particles/mL, yielding a total dose of 3.4 × 10^9^ viral particles). All animals on study received daily ganciclovir (prodrug) treatment (50 mg/kg) intraperitoneally for 4 days, beginning 1 day after CAN-2409/vehicle control administration. Anti–CTLA-4 (clone 9H10; RRID: AB_10950184) and isotype control antibodies were purchased from Bio X Cell and administered intraperitoneally (200 μg diluted in PBS) every 3 days, beginning on the same day as CAN-2409 treatment with three doses total. Tumor growth inhibition rate was calculated by 100 − (ΔT/ΔC × 100), where ΔT is the volume change for all tumors, and ΔC is the average volume change for control tumors.

### Cell isolation

At study endpoint, tumors were cut into small pieces and incubated with collagenase D (1 mg/mL) and DNase I (0.1 mg/mL) in RPMI media (1.2 mL total volume) on a thermomixer for 20 minutes at 37°C. After incubation, samples were filtered through a 70-μm strainer to remove undigested debris. Tumor dLN (i.e., left inguinal LN) was dissected, cleaned, treated in RPMI 1640 medium, and crushed through a 70-μm filter. Whole blood was collected and processed to plasma. Red blood cells were then lysed in the remaining cell pellet, and circulating immune cells were frozen down for downstream analysis.

### Flow cytometry

Cells isolated from tumors and tumor dLNs were resuspended in FACS staining buffer (2% FBS and 2 mmol/L EDTA in PBS) and subjected to Fc block with anti-CD16/32 (1:200, clone 2.4G2; BioLegend, RRID: AB_312800) diluted in FACS staining buffer on ice for 10 minutes before staining with LIVE/DEAD Fixable dead cell stain kits (1:1,000, L34960; Thermo Fisher Scientific), and surface markers diluted in FACS staining buffer on ice for 30 minutes. Cells were then fixed with BD Cytofix Fixation Buffer (554655; BD Biosciences) for 40 minutes and stained for intracellular markers diluted in eBioscience permeabilization buffer (00-8333-56; Thermo Fisher Scientific) at room temperature overnight. To identify Ag-specific CD8^+^ T cells, the cell suspension was incubated with Allophycocyanin-labeled MC38 neo-Ag pentamer H-2Kb-KSPWFTTL (F828-4A-G; ProImmune) diluted to 1:10 in staining buffer for 1 hour at 37°C prior to surface marker staining. To assess the absolute cell numbers, 1 × 10^4^ counting beads (ACBP-100-10; Spherotech) were added to each stained sample at the last step. Data were acquired on the BD LSRFortessa X-20 (BD Biosciences) using FACSDiva 8.0.2 software (BD Biosciences).

The antibiotics raised against the following mouse, Ags were used: CD3 BUV737 (1:200; clone 17A2; BD Biosciences, RRID: AB_2870130), CD4 BV711 (1:200; clone RM4-5; BioLegend, RRID: AB_11219396), CD8a BV510 (1:200; clone 53-6.7; BioLegend, RRID: AB_2561389), CD44 BV785 (1:200; clone IM7; BioLegend, RRID: AB_11218802), Foxp3 e450 (1:200, clone FJK-16s; eBioscience, RRID: AB_1518812), CX3CR1 BV650 (1:200; clone SA011F11; BioLegend, RRID: AB_2565999), CD45.2 BUV395 (1:200; clone 104; BD, RRID: AB_2738867), GZB B AF700 (1:200; clone QA16A02; BioLegend, RRID: AB_2728388), Ki67 PE-Cy7 (1:200; clone SolA15; eBioscience, RRID: AB_11220070), Ly-108 BUV395 (1:200; clone 13G3; BD, RRID: AB_2743205), and PD-1 BV605 (1:200; clone 29F.1A12; BioLegend, RRID: AB_11125371). Gating schemes for analysis of tumor and tumor dLN are shown in Supplementary Figs. S4 and S5.

### Statistical analysis

For all continuous data, normality was determined by the Shapiro–Wilk test. The ANOVA model was used to compare the normal-distributed data. For binary data, Fisher exact test was used. All statistical tests will be at the 0.05 level of significance (*P* < 0.05), *n* ≥ 3, and denoted as follows: *, *P* < 0.05; **, *P* < 0.01; ***, *P* < 0.001; and ****, *P* < 0.0001. Wherever a *P* value is not indicated, no statistical difference was observed. Statistical analysis was performed using GraphPad Prism.

### Data availability

The data generated in this study are available upon request from the corresponding author.

## Results

### CAN-2409 induces tumor regression and T-cell infiltration in the TME

**Figure 1:**
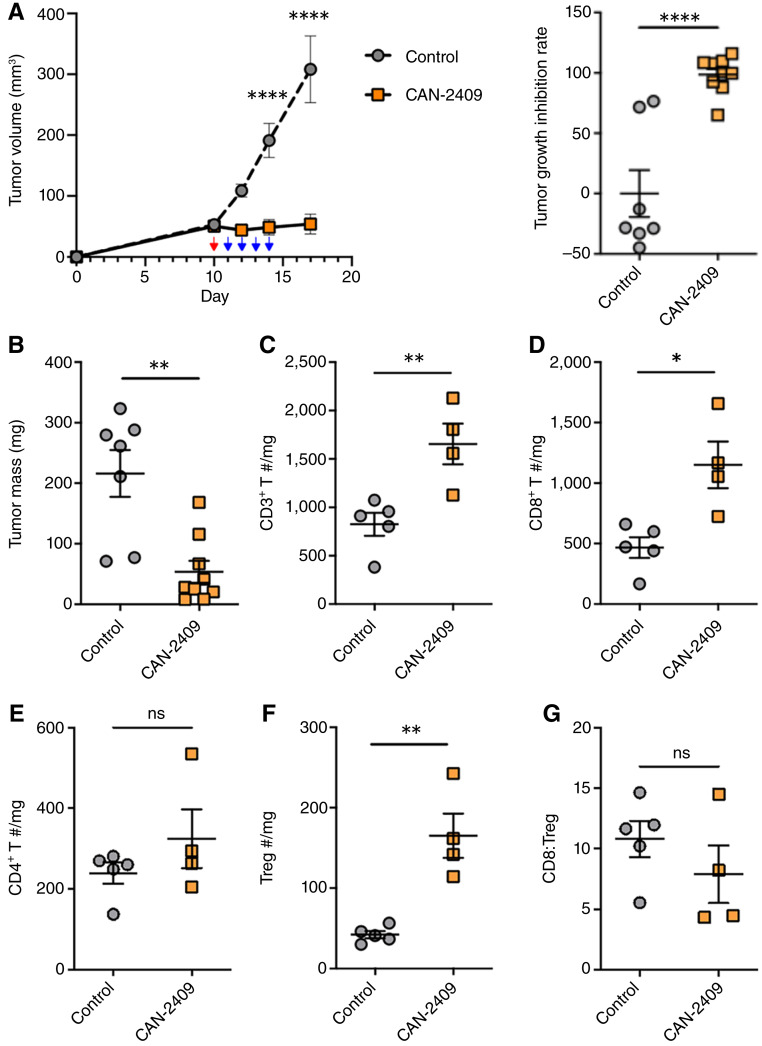
CAN-2409 + prodrug inhibits tumor growth and increases lymphocyte infiltration in MC38 tumor–bearing C57BL/6 mice. **A,** MC38 tumor–bearing mice were treated with or without CAN-2409 i.t. (red arrow) followed by 4 days of i.p. administration of prodrug (blue arrows), and tumor growth was monitored. Left, tumor growth curves. Right, tumor growth inhibition rate after treatment. **B,** Endpoint analysis of tumor mass. **C–G,** Endpoint analysis tumor immune populations assessed by flow cytometry. *N* = 7–9 mice per group (some tumors are too small to be included in the immune cell analysis). Two-tailed *t* test, *, *P* < 0.05; **, *P* < 0.01; ****, *P* < 0.0001.

To investigate the antitumor T-cell response generated by CAN-2409 treatment, we first assessed tumor growth in MC38 tumor–bearing WT mice and profiled tumor-infiltrating lymphocytes (TIL) at the endpoint. MC38 tumor–bearing WT mice were treated with CAN-2409 intratumorally (3.4x10^9^ virus particles) or vehicle (control). All animals (control and CAN-2409 groups) then received prodrug (ganciclovir, 50 mg/kg per day) intraperitoneally on the following 4 days. Prodrug treatment has no effect on tumor growth in preclinical models (9) and thus, providing in combination with vehicle a suitable control for *in vivo* experiments. Tumor growth measurements were recorded daily. Mice were euthanized 17 days after inoculation. Robust tumor growth inhibition was observed after i.t. administration of CAN-2409 followed by prodrug (Fig. 1A; Supplementary Fig. S1). At the endpoint, tumors from CAN-2409 + prodrug–treated mice were significantly smaller than those from controls (53.6 ± 18.3 mg in CAN-2409–treated mice vs. 216.0 ± 38.8 mg in control mice; *P* = 0.001; Fig. 1B). As expected, there was significant infiltration of CD3^+^ T cells in CAN-2409–treated animals (Fig. 1C), the majority of which were CD8^+^ T cells (1,151.2 ± 193.0 cells/mg of tissue in CAN-2409–treated mice vs. 468.1 ± 85.4 cells/mg of tissue in control mice; *P* = 0.01; Fig. 1D). Notably, a significant increase in the number of regulatory T cells (Treg) was identified in CAN-2409–treated mice (Fig. 1E and F); as a result, the CD8:Treg ratio was similar among treatment groups (Fig. 1G).

### CAN-2409 treatment induces expansion of T cells within the TME

**Figure 2:**
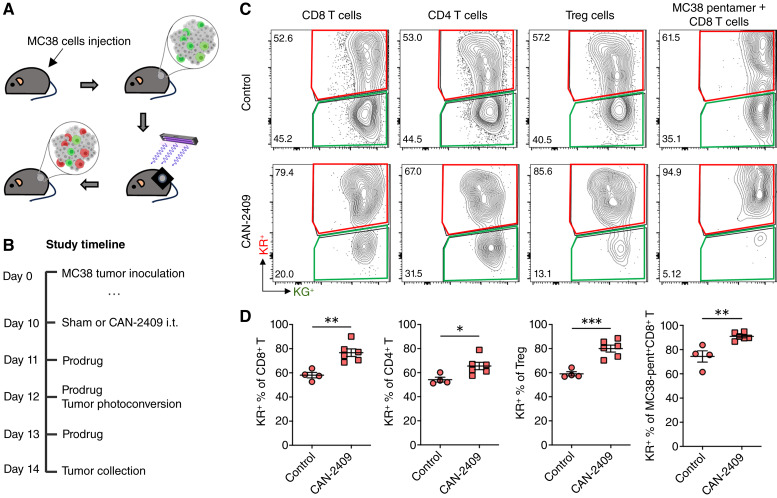
CAN-2409 + prodrug induces expansion of immune cells and neoantigen-specific T cells retained in tumors. **A,** Summary of MC38 implanted subcutaneous on the flank of C57BL/6 Kaede mice and photoconverted by violet light exposure. **B,** Study timeline for experiments assessing the effect of CAN-2409 + prodrug on TME modulation in Kaede photoconvertible mice. **C,** Representative flow cytometry plots showing KR^+^ and KG^+^ expression in immune subpopulations within tumors. MC38 pentamers were used to identify antigen-specific CD8^+^ T cells. **D,** Quantification of newly entering (KG^+^) cells for each T-cell subset. *N* = 4–6 mice per group. Two-tailed *t* test, *, *P* < 0.05; **, *P* < 0.01; ***, *P* < 0.001.

Next, we investigated whether the increased number of T cells within the tumor after CAN-2409 treatment was due to proliferation of resident cells or due to increased cell migration. Kaede photoconvertible mice were engrafted with MC38 tumors to enable dynamic labeling of the tumor tissue. All cells in Kaede mice express a GFP that is converted to a red fluorescent version upon exposure to violet light. Therefore, in tumor-bearing Kaede mice, the immune infiltrate is Kaede Green (KG^+^). Upon photoconversion specifically of the tumor, almost all the immune cells within the tumor are converted to Kaede Red (KR^+^) (see “Materials and Methods”). This enables a window of time, whereby newly entering immune cells (KG^+^) can be distinguished from cells retained within the tumor at the time of photoconversion, allowing the assessment of immune cell recruitment versus retention (Fig. 2A). Importantly, CAN-2409 + prodrug treatment exerts comparable tumor growth inhibition in MC38 tumor–bearing Kaede mice compared with WT mice 4 days after CAN-2409 administration (Supplementary Fig. S2A), confirming the utility of the Kaede model for mechanistic studies. Owing to the mechanism of action for CAN-2409 is fully dependent on CD8^+^ T cells (5–7), the dynamic tracking analysis of immune cells retained and recruited to the TME was focused on T-cell subsets.

MC38 tumor–bearing Kaede transgenic mice were treated with vehicle or CAN-2409 i.t., and then prodrug was given on the three following days. The tumor was photoconverted 2 days after CAN-2409 administration, and tumors were collected 48 hours later, a time point selected based on immune cell recruitment dynamics in this model (28) that allows the tumor immune response to evolve prior to analysis (Fig. 2B). CAN-2409 treatment resulted in a significant increase in the proportion of KR^+^ cells compared with control animals, including CD8^+^ (76.7% ± 3.1% vs. 58.1% ± 2.3%; *P* = 0.003) and CD4^+^ T cells (65.6% ± 3% vs. 54.2% ± 2%; *P* = 0.03), Tregs (80% ± 2.9% vs. 59% ± 1.7%; *P* = 0.001), and neoantigen-specific CD8^+^ T cells (MC38-pentamer^+^CD8^+^ T cells, 90.9% ± 1.2% vs. 74.4% ± 4.7%; *P* = 0.003; Fig. 2C and D). Although a modest decrease in the absolute number of CD4^+^ T cells per mg tumor was observed at this timepoint (407.9 ± 34.3 vs. 190.9 ± 58.3; *P* = 0.024), we observed no changes in the absolute number of CD8^+^ T cells, Tregs, or neoantigen-specific CD8^+^ T cells per mg tumor (Supplementary Fig. S2B). Together these data suggest that the changes observed in the proportion of KR^+^ and KG^+^ cells are not driven by changes in the total number of cells per mgs of tumor at this timepoint (4 days after CAN-2409 administration), and that the immune composition of the TME likely continues to evolve over time as the antitumor immune response progresses (e.g., Fig. 1 at 7 days after CAN-2409 administration). Collectively, these data indicate that treatment with CAN-2409 + prodrug results in the expansion of immune cells within the TME rather than a clear increase in the number of T cells recruited into the tissue.

**Figure 3:**
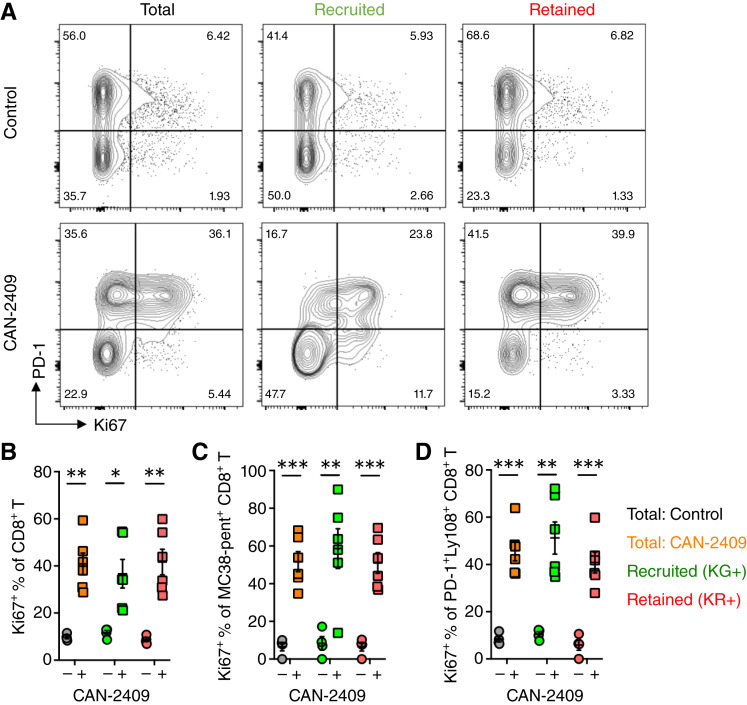
Expansion of immune cells within tumors is, in part, due to CAN-2409 + prodrug–mediated stimulation of proliferation of T-cell subsets in the TME (total, neoantigen-specific, and stem-like CD8^+^ T cells). **A,** The effect of CAN-2409 + prodrug treatment on proliferative status of T-cell subsets was assessed using Ki67 as a marker. Representative flow cytometry plots for total, KG^+^, and KR^+^ populations of total, TAA–specific (MC38 pentamer^+^), and stem-like (PD-1^+^Ly108^+^) CD8^+^ T cells and quantification are shown in **B–D**. *N* = 4–6 mice per group. Two-tailed *t* test, *, *P* < 0.05; **, *P* < 0.01; ***, *P* < 0.001.

To further investigate how CAN-2409 + prodrug treatment increased the number of T cells within the tumor, we focused on the CD8^+^ T-cell compartment and assessed Ki67 expression as a measure of proliferation. Considering the total CD8^+^ T-cell compartment, CAN-2409 treatment clearly increased the proportion of CD8^+^ T cells expressing Ki67 (total T cell: 40.9% ± 4.6% vs. 9.7% ± 0.7%; *P* = 0.001; Fig. 3A and B). Analysis of the cells recently recruited into the tumor (KG^+^) versus those retained within the tumor (KR^+^) revealed a significant increase in the proportion of Ki67^+^ CD8^+^ T cells in both subsets (KG^+^: 36.7% ± 6% vs. 11.6% ± 1%; *P* = 0.01; KR^+^: 41.7% ± 5.4% vs. 8.7% ± 0.8%; *P* = 0.001; Fig. 3A and B). Using MC38 pentamers to track an antigen-specific population of CD8^+^ T cells, a comparable increase in the proportion of Ki67^+^ cells was observed by both KG^+^ and KR^+^ T cells (KG^+^: 58.6% ± 10.5% vs. 8.4% ± 3.5%; *P* = 0.006; KR^+^: 51% ± 5.5% vs. 6.6% ± 2.3%; *P* = 0.0002, Fig. 3C). We also specifically assessed PD-1^+^ Ly108^+^ expression to visualize the “stem-like” progenitor population and thought to sustain the exhausted compartment and, again, observed increased Ki67 expression in CAN-2409 + prodrug–treated mice (total T cell: 45.8% ± 4.2% vs. 8.4% ± 1.2%; *P* = 0.0001; Fig. 3D). Together, these data indicate that CAN-2409 + prodrug treatment drives changes in the TME that make it more supportive (or less restrictive) of CD8^+^ T-cell proliferation, potentially explaining the increase in CD8^+^ T cells in the tumor.

**Figure 4:**
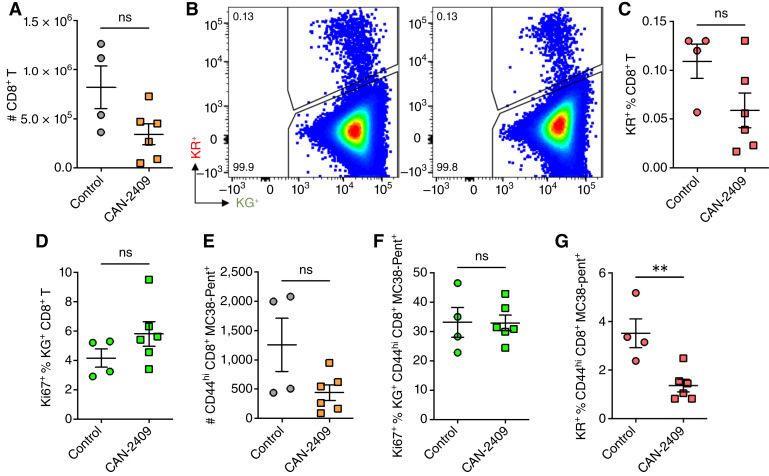
No significant changes of CD8^+^ T cell in the dLN following CAN-2409 treatment. **A,** Quantification of total CD8^+^ T cell in the tumor dLN. **B,** Representative flow cytometry plots for KG^+^ and KR^+^ populations of CD8^+^ T cells and quantification are shown in **C** for KR^+^ CD8^+^ T cells. **D,** Quantification of Ki67^+^ percentage in the KG^+^CD8^+^T cells. **E,** Quantification of total TAA–specific (CD44^hi^ MC38 pentamer^+^) CD8^+^ T cell in the tumor dLN. **F,** Quantification of Ki67^+^ percentage in TAA–specific KG^+^CD8^+^T cells. **G,** KR^+^ percent of TAA–specific (MC38 pentamer^+^) CD8^+^ T cell. All data are measured by flow cytometry. *N* = 4–6 mice per group. Two-tailed *t* test, **, *P* < 0.01.

We further assessed whether differences in antitumor T-cell proliferation were specific to responding cells within the tumor or also evident within the dLN. Analysis of the total CD8^+^ T-cell numbers in the dLN revealed no significant increase in CAN-2409 + prodrug–treated mice (Fig. 4A). Photoconversion of the tumor allowed for the identification of CD8^+^ T-cell trafficking from the labeled tumor to the dLN such that they could be specifically excluded from analysis of cells responding within the dLN. No significant difference in the proportion of labeled (KR^+^) CD8^+^ T cells in the dLN was shown (Fig. 4B and C). Analysis of Ki67 expression of KG^+^ CD8^+^ T cells in the dLN also revealed no difference, indicating that CAN-2409 + prodrug treatment did not increase T-cell proliferation in the dLN (Fig. 4D). Rather, we observed a trend toward a lower percentage of KR^+^ CD8^+^ T cells, which trafficked from the tumor to the dLN at this timepoint (4 days after CAN-2409 treatment and 2 days after photoconversion). To assess this in more detail, we analyzed the number of TAA-specific CD8^+^ T cells (CD44^+^ pentamer–specific) in the dLN and observed no significant change in cell numbers (Fig. 4E) or in the percentage of these cells that expressed Ki67 (Fig. 4F). Interestingly, we did find that the proportion of KR^+^ TAA-specific CD8^+^ T-cell trafficking from the tumor to the dLN was significantly reduced after CAN-2409 + prodrug treatment, suggesting that actively responding T cells were retained within the tumor (1.4% ± 0.3% vs. 3.5% ± 0.6%; *P* = 0.005; Fig. 4G). In summary, these data suggest that the increased in the number of CD8^+^ T cells observed in tumors treated with CAN-2409 at day 7 (Fig. 1D) is more likely to reflect the enhanced T-cell proliferation observed in the TME in the early phases of the antitumor response rather than late recruitment of T cells. This hypothesis is also supported by the low number of CD8^+^ T cells observed in the dLN, suggestive of early retention of activated cells in the tumor followed by expansion of effector cells at this site but not in the dLN.

### CAN-2409 treatment supports reinvigoration of TAA-specific CD8^+^ T cells in the TME and expansion of less-activated Tregs

**Figure 5:**
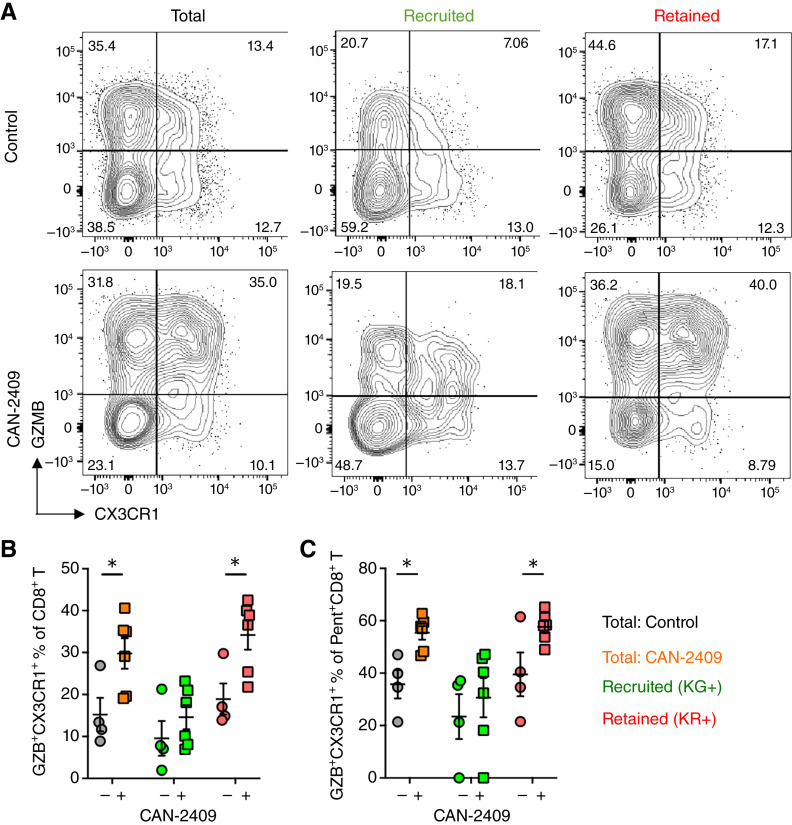
CAN-2409 + prodrug treatment reinvigorates tumor-retained, exhausted CD8^+^ T cells, and tumor-experienced CD8^+^ T cells, which egress tumors, have enhanced functionality. The effect of CAN-2409 + prodrug treatment on terminal exhaustion of CD8^+^ T cells was assessed in the tumor using CX3CR1 as a marker coupled with the granzyme B expression. **A,** Representative flow cytometry plots for total, KG^+^, and KR^+^ populations of total and TAA-specific (MC38 pentamer^+^) CD8^+^ T cells and quantification are shown in **B**. *N* = 4–6 mice per group. Two-tailed *t* test, *, *P* < 0.05.

Terminal exhaustion of the CD8^+^ T-cell compartment is a common mechanism underpinning resistance to immunotherapy. To investigate the effect of CAN-2409 on CD8^+^ T-cell exhaustion, we evaluated TILs and peripheral cells from CAN-2409–treated mice for the expression of CX3CR1, which is retained on intermediate-exhausted but lost on terminally exhausted CD8^+^ T cells (29–31). Alongside CX3CR1, we also assessed GZB as an effector mechanism mediating tumor-cell lysis. We observed that treatment with CAN-2409 induced a striking increase in the percentage of GZB^+^CX3CR1^+^ cells within the total CD8^+^ T-cell compartment (Fig. 5A). Dividing these cells into KG^+^ and KR^+^ subsets revealed that the increased proportion of GZB^+^CX3CR1^+^ cells was most evident within the KR^+^ population, consistent with reinvigoration of CD8^+^ T cells present within the tumor at the time of photolabeling (KR^+^: 34.2% ± 3.5% vs. 18.9% ± 3.7%; *P* = 0.02; Fig. 5A and B). Comparable findings were observed when TAA-specific CD8^+^ T cells were assessed (KR^+^: 57.6% ± 2.3% vs. 39.5% ± 8.3%; *P* = 0.035; Fig. 5C). Thus, CAN-2409 + prodrug treatment results in enhanced proliferation of CD8^+^ T cells within the remodeled TME, and this is accompanied by differentiation into an intermediate-exhausted state that retains key effector functions contributing to increased tumor control.

**Figure 6:**
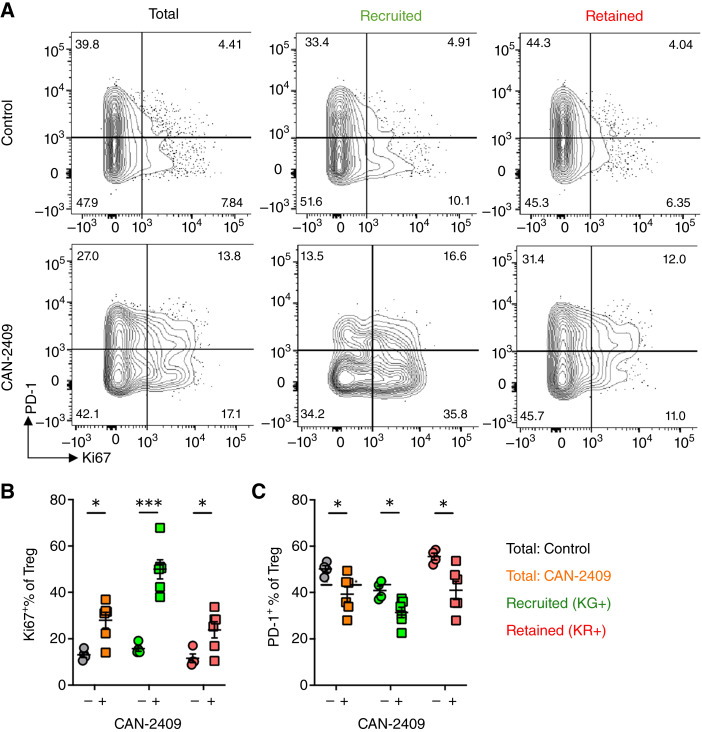
Tumor-recruited, tumor-retained, and tumor-experienced Tregs, which egress tumors, adopt an altered phenotype after CAN-2409 + prodrug treatment. The effect of CAN-2409 + prodrug treatment on proliferative status of Treg cells was assessed using Ki67 as a marker coupled with the PD-1 expression. **A,** Representative flow cytometry plots for total, KG^+^, and KR^+^ Treg cells. Quantification of tumor experiencing Treg (KR^+^) for proliferation status (Ki67), **B**, and immune suppressive functional markers (PD-1, **C**, *N* = 4–6. Two-tailed *t* test, *, *P* < 0.05; ***, *P* < 0.001.

In addition to the enhanced CD8^+^ T-cell response, CAN-2409 + prodrug treatment resulted in increased numbers of Tregs within the tumor (Fig. 1). Given the improved tumor control observed in treated mice, we investigated why Tregs failed to impede the CD8^+^ T cells within CAN-2409–treated tumors. Analysis of the Treg compartment revealed a significant increase in the proportion of Ki67^+^ Tregs (Fig. 6A). Interestingly, this was most apparent within the KG^+^ cells, suggesting that the increased number of Tregs in the tumor results from expansion of these cells within the TME (KG^+^ CAN-2409: 50% ± 4.2% vs. KG^+^ control: 15.8% ± 1.2%; *P* = 0.0002; Fig. 6A and B). Furthermore, we found a significant decrease in the proportion of PD-1^+^ Tregs, consistent with a less-activated Treg compartment after CAN-2409 treatment (total CAN-2409: 39.3% ± 3.3% vs. total control: 50.2% ± 1.4%; *P* = 0.03; Fig. 6A and C). Collectively, these data suggest that CAN-2409 treatment results in an expanded and more functional CD8^+^ compartment within the tumor that improves tumor control. This is accompanied by an increase in Tregs, but these are of an altered phenotype and may fail to impede the enhanced CD8^+^ T-cell response.

### CAN-2409 + prodrug treatment combined with anti–CTLA-4 antibody therapy improves tumor control

Resistance or loss of response to ICI is common, and combination of multiple lines of systemic immunotherapy does not necessarily improve efficacy and is often complicated by systemic toxicity. We have previously demonstrated that CAN-2409, when added to anti-PD(L)1 antibody treatment, extends survival in preclinical tumor models (7) and changes the trajectory of tumor growth and reinvigorates the antitumoral response in patients inadequately responding to anti-PD(L)1 alone (21, 22). In the current study, we aimed to evaluate potential synergy of our agent with anti–CTLA-4 antibody therapy in MC38 tumor–bearing mice to provide proof-of-concept for additional potential clinical studies in patients.

**Figure 7:**
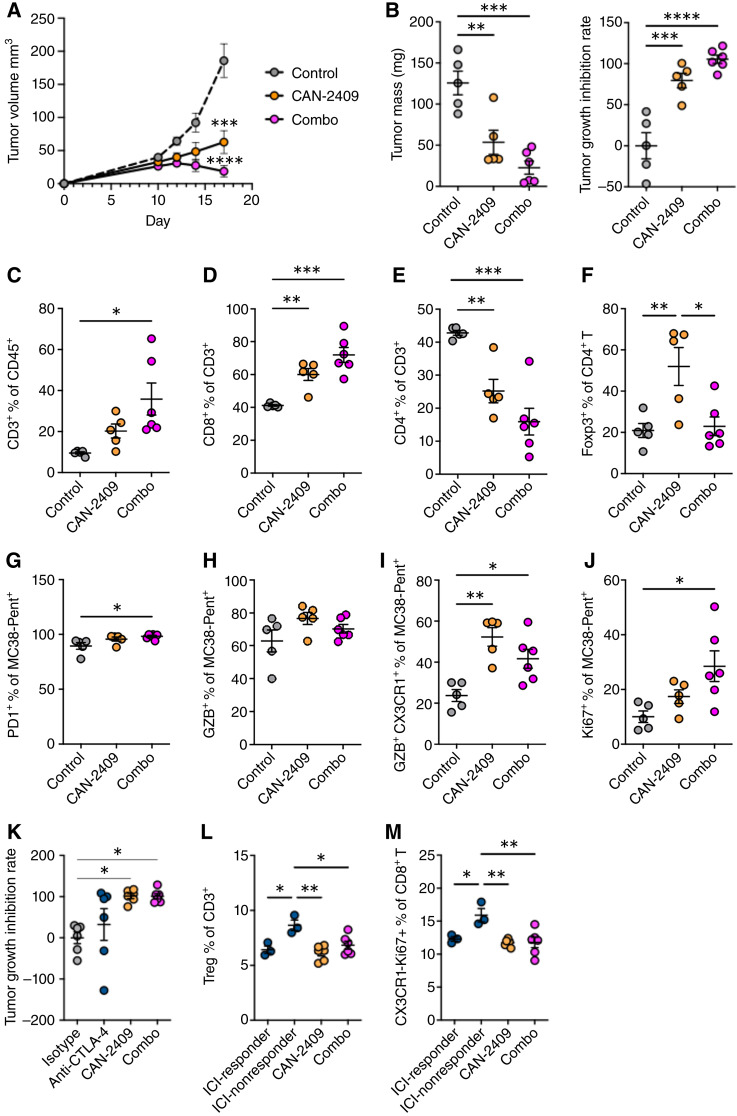
Tumor growth was better controlled when CAN-2409 + prodrug treatment was combined with anti–CTLA-4 antibody therapy compared with CAN-2409 + prodrug alone. **A,** MC38 tumor–bearing mice were treated with or without CAN-2409 i.t. at day 10, followed by 4 days of i.p. administration of prodrug. Anti–CTLA-4 or isotype control were treated i.p. on day 10, 13, and 16. Tumor growth was monitored, and tumors were collected on day 17. **B,** Tumor weight quantification (left) and tumor growth inhibition rate (right) at study endpoint. Quantification of CD3^+^ cells (**C**), CD8^+^ T cells (**D**), CD4^+^ T cell (**E**) and Treg (**F**) by flow cytometry. **G** and **H,** Characterization of TAA-specific CD8^+^ T cells for PD1^+^ (**G**), GZB^+^ (**H**), GZB^+^CX3CR1^+^ (**I**) and Ki67^+^ (**J**). **K,** MC38tumor–bearing mice were treated with or without CAN-2409 i.t. at day 8, followed by 3 days of i.p. administration of prodrug. Anti–CTLA-4 or isotype control was treated i.p. on day 8, 11, and 14. Tumor growth was monitored, and tumors were collected on day 18. Tumor growth inhibition after treatment was calculated. Quantification of Treg (**L**) and CX3CR1^-^Ki67^+^ CD8^+^ T cells (**M**) in tumor dLNs. *N* = 5–6 mice per group. One-way ANOVA with Tukey correction, *, *P* < 0.05; **, *P* < 0.01; ***, *P* < 0.001; ****, *P* < 0.0001.

Tumor growth was better controlled when CAN-2409 treatment was combined with anti–CTLA-4 antibody therapy compared with CAN-2409 + prodrug treatment alone (Fig. 7A and B; Supplementary Fig. S3A). At the endpoint, tumors from combination therapy–treated mice were significantly smaller than controls (22.8 ± 6.5 mg in combo vs. 53.6 ± 10.9 mg in CAN-2409 + prodrug alone and 125.7 ± 12.2 mg in control mice; *P* = 0.0001; Fig. 7B). Combination therapy also resulted in a significant increase in the proportion of CD8^+^ T cells within tumors compared with CAN-2409 + prodrug treatment alone (71.9% ± 3.7% in combo vs. 60.1% ± 2.7% in CAN-2409 + prodrug alone and 41.2% ± 0.4% in control mice; *P* = 0.0001; Fig. 7C and D; Supplementary Fig. S3B). At the same time, combination therapy reduced the proportion of Tregs within the CD4^+^ T-cell compartment, induced by CAN-2409 + prodrug treatment, back to control levels (22.9% ± 3.7% in combo vs. 52.0% ± 6.8% in CAN-2409 + prodrug alone and 20.9% ± 2.9% in control mice; *P* = 0.01; Fig. 7E and F; Supplementary Fig. S3B). Analysis of TAA-specific CD8^+^ T cells revealed that combination therapy increased the proportion of PD-1^+^ cells compared with CAN-2409 + prodrug alone, although levels were high in all treatment groups (Fig. 7G). Compared with the control group, there was a trend toward increased expression of GZB in TAA-specific CD8^+^ T cells as both CAN-2409 + prodrug monotherapy and the combination of CAN-2409 + prodrug with anti–CTLA-4 antibody (Fig. 7H) and significantly increased the proportion of intermediate exhausted (CX3CR1^+^GZB^+^) T cells (41.7% ± 3.8% in combo vs. 52.3% ± 3.4% in CAN-2409 + prodrug alone and 23.7% ± 2.5% in control mice; *P* = 0.02; Fig. 7I). Notably, the combined treatment induced a higher proportion of Ki67+ cells within the TAA-specific CD8^+^ T cells (28.5% ± 4.6% in combo vs. 17.4% ± 1.8% in CAN-2409 + prodrug alone and 10.0% ± 1.8% in control mice; *P* = 0.02; Fig. 7J).

A subsequent study was performed to define effects that could be attributed to anti–CTLA-4 antibody therapy or CAN-2409 alone in our model. Variable responses to anti–CTLA-4 antibody treatment alone were observed; tumor growth was controlled in 50% of the MC38 tumor–bearing mice with the remaining 50% of mice having tumor growth comparable to control (Fig. 7K). CAN-2409 treatment resulted in significant tumor growth inhibition, comparable to combined CAN-2409/anti–CTLA-4 antibody treatment (Fig 7K). Given the variability of the responses to anti–CTLA-4 antibody therapy, we aimed to analyze the biological responses associated with “responders” and “nonresponders” (responders: tumor growth inhibition rate above median, nonresponders: tumor growth inhibition rate below median, median = 72.3%). Mice included in the nonresponder to anti–CTLA-4 antibody group presented elevated Treg numbers as compared with responders (Fig. 7L) and high frequency of nonactivated yet proliferating CD8 T cells (CX3CR1-Ki67^+^CD8^+^) in their dLNs (Fig. 7M). Surprisingly, the increase in these cell populations was abrogated when anti–CTLA-4 antibody therapy was used in combination with CAN-2409. Together, these data suggest that the combination therapy consisting of CAN-2409 + prodrug treatment and anti–CTLA-4 antibody therapy improved tumor control beyond that observed with CAN-2409 + prodrug alone and CTLA-4 alone, which could be explained by simultaneously enhancing the CD8^+^ T-cell response and impeding accumulation of Tregs.

## Discussion

Multiple studies have previously demonstrated antitumor activity of CAN-2409 plus prodrug without significant toxicity in a variety of animal models of cancer, including brain, breast, ovarian, lung, esophageal, and prostate cancer (5, 6, 9, 32–34). These experiments, supported by observations in experimental clinical trials of CAN-2409 in patients with cancer, have shown that the mechanism of action of CAN-2409 is largely dependent on CD8^+^ T cells that are reprogrammed after treatment. However, the exact mechanism as to how i.t. CAN-2409 treatment results in a systemic antitumor response and the interplay between antitumor and antiviral immune activation after treatment is still incompletely understood. Building on the findings in traditional syngeneic tumor models, in this study, we exploited the dynamic cell tracking capability of Kaede transgenic mice to clarify how CAN-2409 + prodrug alters the antitumor T-cell response. The results presented in this study provide insight into the evolution of the immune response from local changes in the TME after local CAN-2409 injection plus prodrug treatment into systemic antitumor immunity.

Using a photoconvertible mouse tumor model that enables dynamic tracking of responding immune cells, we characterized the dynamics of the T-cell response within treated tumors and tumor dLNs after CAN-2409 treatment in a murine model of colorectal cancer. CAN-2409 treatment resulted in reduced tumor volume and changes in the TME. We demonstrated that CD8^+^ T cells infiltrate the tumors and expand locally in the TME after treatment, resulting in expanded intermediate-exhausted T cells able to control tumor growth. These observations support earlier histologic findings, demonstrating expansion of the CD8^+^ T-cell population and closer proximity between cytotoxic CD8^+^ TILs and tumor cells in the TME after CAN-2409 treatment in patients with NSCLC, consistent with improved antitumor immunity.

It has previously been suggested that an increase in tumor dwell time (length of time spent by immune cells in the TME) induces functional changes in multiple immune cell types including CD8^+^ T cells (28), NK cells (26), and subsets of dendritic cells (27). Longer dwell times have been associated with a transition to an “exhausted” state characterized by transcriptional changes, increased expression of inhibitory receptors, and reduced effector function (35). Our data indicate that CAN-2409 treatment enhances immune cell retention within the tumor and, likely, tumor dwell time for all T-cell subsets assessed. Moreover, mirroring the retention of immune cells within the TME, subsets of CD8^+^ T cells were observed at lower levels in the tumor dLN, which could be both the result of increased tumor dwell time and changes in the rate at which these cells traffic into and out of the dLN, as described by others (28, 35). Notably, CAN-2409–mediated remodeling of the TME was associated with increased functionality, including expansion of TAA-specific CD8^+^ T cells, supporting their critical role in the efficacy of a viral immunotherapy like CAN-2409. This improved function, despite the indication of increased tumor dwell time, is likely to result from the exposure of the retained immune cells to the highly inflammatory microenvironment established by CAN-2409 treatment. Importantly, the combination of increased migration, enhanced antigen exposure, and upregulation of inflammatory signals in the TME results in an increase in stem-like features in CD8^+^ T cells, which is critical for long-term maintenance of antitumor T-cell responses (36). This effect, which likely supports the development of a durable local and systemic antitumor immune response, is in line with evidence of CD8^+^ T-cell activation, proliferation, and expansion of memory T cells, as observed in patients after CAN-2409 treatment (21, 22).

It is well established that important counterbalances are in place to limit an overexuberant T-cell response, and these regulatory mechanisms are often highjacked by tumors to limit antitumor immunity (37). Among those, Treg cells are known to constrain cytotoxic T-cell responses through multiple mechanisms. In the initial immunophenotyping of tumors from CAN-2409–treated mice, we observed an increase in the number of Tregs in the tumors concomitant to the increase in total CD8^+^ T cells, but there was no change in the ratio of CD8^+^ T cells to Tregs. Interestingly, using tumor-bearing Kaede mice, we also observed an altered phenotype of newly recruited Treg cells. In CAN-2409–treated tumors, but not in controls, Tregs were characterized by enhanced proliferation and decreased expression of the immune checkpoints PD-1 and Inducible costimulator. Notably, previously published scRNA-seq analysis of heterogeneity within newly entering and resident TILs in MC38 tumor–bearing Kaede mice showed costimulatory/coinhibitory receptors, including PD-1, are elevated on Tregs over time, and those with the highest levels have been had the longest tumor dwell time and resemble highly activated Tregs described by others (28). Tregs retained and recruited into tumors of CAN-2409–treated mice and thus appear to be less activated or mature than Tregs in control tumors.

Immune checkpoint blockade has emerged as a foundational immunotherapy strategy, yet not all patients respond and loss of efficacy after initial response is also observed. More recently, investigation of these agents in combination with therapeutics displaying complementary or synergistic mechanisms of action has been explored. Benefits associated with the combination of CAN-2409 + prodrug with anti–PD-(L)1 checkpoint inhibitors have been described in both preclinical and clinical settings (refs). However, combination therapy with other immune checkpoints has not been investigated. CTLA-4 is expressed on several cell types within the TME, including Tregs. Moreover, several lines of evidence indicate that anti–CTLA-4 antibody therapy can constrain the expansion of the Treg cell, shifting their phenotype to a less suppressive state and regulating the crosstalk between Tregs and CD4^+^ T cells (38–40). These data combined with our observation on the activity of CAN-2409 on the Treg population suggest a potential synergy between these two agents. Indeed, use of the addition of anti–CTLA-4 antibody therapy to CAN-2409 treatment resulted in better controlled tumor growth compared with CAN-2409 treatment alone. This effect could be explained in part by the enhanced CD8^+^ T-cell–mediated effector function and diminished immunosuppression, as shown by changes in the tumor and tumor dLN Treg compartment. Together this data support the exploration of this combination treatment in tumors with primary resistance to anti–CTLA-4 antibody treatment. Combination immunotherapy has been an area of active investigation over the last decade. Given the relatively small number of patients who have durable responses to ICI therapies, identification of novel approaches for combination with these agents is of critical importance. Dynamic responses to anti–PD-L1 antibody therapy alone have already been well characterized using tumor-bearing Kaede transgenic mice. Anti–PD-L1 therapy has been demonstrated to induce reinvigoration of tumor CD8^+^ T cells and enhance function of newly entering effector cells (28). Enrichment of an activated state of tumor-retained mature DCs enriched in immunoregulatory molecules and increased expression of T-cell stimulatory molecules with anti–PD-L1 therapy have also been described (27). CAN-2409 treatment in combination with anti-PD-1 antibody therapy has previously been explored in a murine model of glioblastoma (7) and, more recently, in patients with newly diagnosed high-grade glioma (41). The results presented in this study show the first evidence of synergy for the combination of CTLA-4 with CAN-2409. Given the distinct mechanism of action for anti–PD-(L)1 versus anti–CTLA-4 antibody therapies with regard to the identity and site of the immune cell populations that are affected, particularly the combinatorial effects observed on Tregs in this study, these data suggest that CAN-2409 may have utility in distinct patient populations including those with resistance to anti–CTLA-4 antibody treatment.

Collectively, these data defined at least two temporally distinct pathways underpinning the mechanism of action of CAN-2409 action that overcome cell exhaustion and decreased immune suppression. The results also support the rationale for future clinical trials of CAN-2409 treatment combined with anti–CTLA-4 antibody therapy.

## Supplementary Material

Supplementary Figure S1 Supplemental Figure 1 shows individual tumor growth of animals treated with and without CAN-2409 and prodrug

Supplemental Figure 2 Supplemental Figure 2 shows the tumor growth inhibition rate 4 days after treatment and immune cell quantification in tumor tissue in Kaede mice

Supplemental Figure 3 Supplemental Figure 3 shows the combined effect of CAN-2409 and anti-CTLA-4 Ab treatment on individual tumor growth curves and immune cell quantification in tumor tissue

Supplemental Figure 4 Supplemental Figure 4 shows the flow cytometry gating scheme applied to tumor samples

Supplemental Figure 5 Supplemental Figure 5 shows the flow cytometry gating scheme applied to tumor-draining lymph node samples
