# Supplementary material for: Dynamic Tracking of Tumor Microenvironment Modulation Using Kaede Photoconvertible Transgenic Mice Unveils New Biological Properties of Viral Immunotherapy
Source: Cancer Res Commun. 2025 Feb 17;5(2):327–38. doi: 10.1158/2767-9764.CRC-24-0434 (PMC11831061; doi:10.1158/2767-9764.CRC-24-0434)
Supplement: Supplementary Figure S1 — Supplemental Figure 1 shows individual tumor growth of animals treated with and without CAN-2409 and prodrug [file crc-24-0434_supplementary_figure_s1_suppsf1.pdf]

Supplemental Figure 1

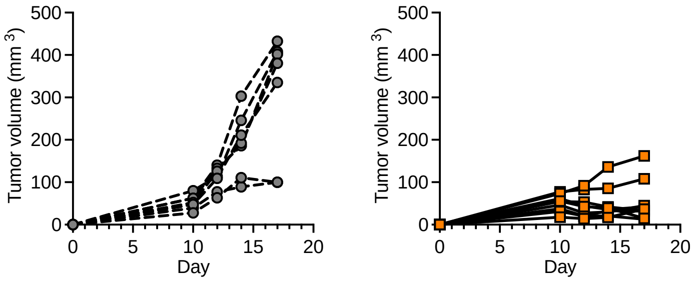

Supplemental Figure 1: Individual tumor growth of animals treated with and without CAN-2409 and prodrug. MC38-tumor bearing mice were treated with or without CAN-2409 i.t. followed by 4 days of i.p. administration of prodrug, and tumor growth was monitored. Left panel: control group. Right panel: CAN-2409 group. N=7-9 mice per group.
