# Supplementary material for: Dynamic Tracking of Tumor Microenvironment Modulation Using Kaede Photoconvertible Transgenic Mice Unveils New Biological Properties of Viral Immunotherapy
Source: Cancer Res Commun. 2025 Feb 17;5(2):327–38. doi: 10.1158/2767-9764.CRC-24-0434 (PMC11831061; doi:10.1158/2767-9764.CRC-24-0434)
Supplement: Supplemental Figure 2 — shows the tumor growth inhibition rate 4 days after treatment and immune cell quantification in tumor tissue in Kaede mice [file crc-24-0434_supplemental_figure_2_suppsf2.pdf]

Supplemental Figure 2

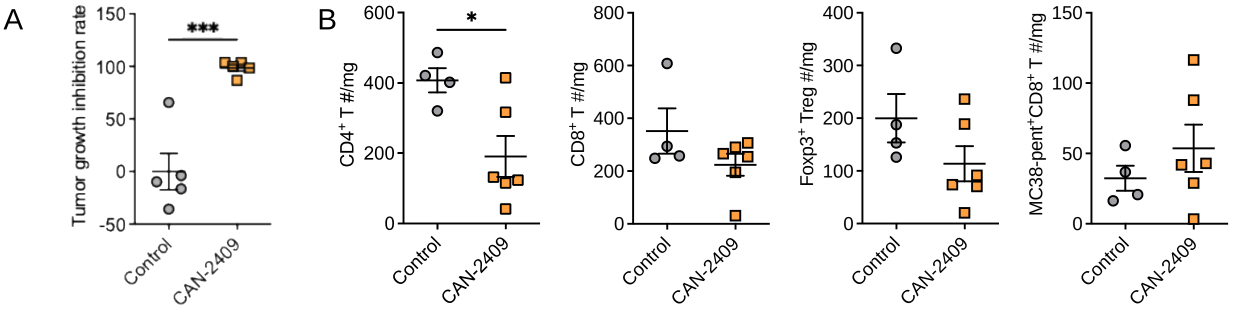

Supplemental Figure 2: Tumor growth inhibition rate 4 days after treatment and immune cell quantification in tumor tissue in Kaede mice. A. Tumor growth inhibition rate in WT vs. Kaede MC38-tumor bearing mice treated with or without CAN-2409 i.t. followed by i.p. administration of prodrug. B. Quantification of total number per mg of tissue for $CD8^+$, MC38-$Pent^+$ $CD8^+$, $CD4^+$ T cells, and Treg in tumors treated with CAN-2409 + prodrug compared to control. N=4-6 mice per group. Two-sided t-test, *, $p < 0.05$.
