# Supplementary material for: Dynamic Tracking of Tumor Microenvironment Modulation Using Kaede Photoconvertible Transgenic Mice Unveils New Biological Properties of Viral Immunotherapy
Source: Cancer Res Commun. 2025 Feb 17;5(2):327–38. doi: 10.1158/2767-9764.CRC-24-0434 (PMC11831061; doi:10.1158/2767-9764.CRC-24-0434)
Supplement: Supplemental Figure 3 — shows the combined effect of CAN-2409 and anti-CTLA-4 Ab treatment on individual tumor growth curves and immune cell quantification in tumor tissue [file crc-24-0434_supplemental_figure_3_suppsf3.pdf]

Supplemental Figure 3

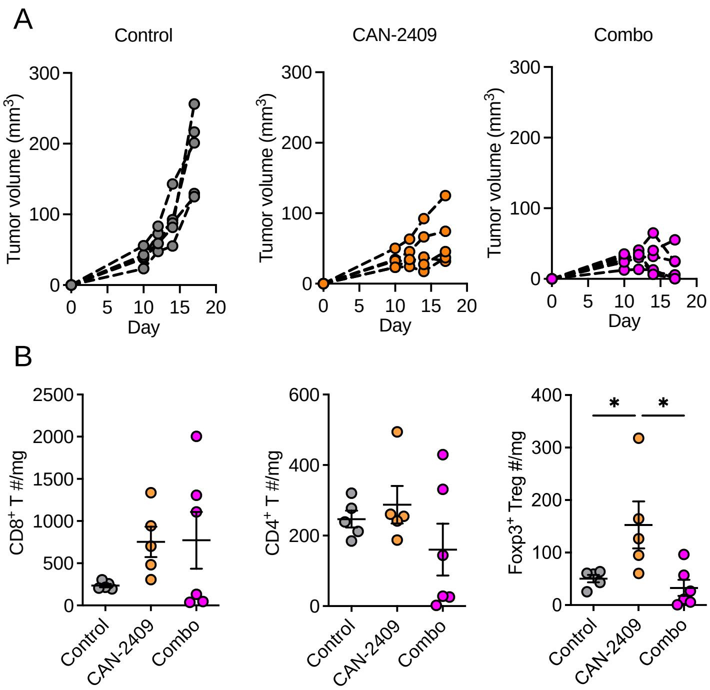

Supplemental Figure 3: Combined effect of CAN-2409 and anti-CTLA-4 Ab treatment on individual tumor growth curves and immune cell quantification in tumor tissue. A. Individual tumor growth curves from MC38-tumor bearing mice were treated with or without CAN-2409 i.t. at day 10, followed by 4 days of i.p. administration of prodrug. Anti-CTLA-4 or isotype control were treated i.p. on day 10, 13 and 16. B. Quantification of total number per mg of tissue for $CD8^+$, $CD4^+$ T cells, and Treg in tumors treated with CAN-2409 + prodrug and with or without anti-CTLA-4 antibody compared to control. N=5-6 mice per group. One-way ANOVA with Tukey correction, *, $p < 0.05$.
