# Supplementary material for: Dynamic Tracking of Tumor Microenvironment Modulation Using Kaede Photoconvertible Transgenic Mice Unveils New Biological Properties of Viral Immunotherapy
Source: Cancer Res Commun. 2025 Feb 17;5(2):327–38. doi: 10.1158/2767-9764.CRC-24-0434 (PMC11831061; doi:10.1158/2767-9764.CRC-24-0434)
Supplement: Supplemental Figure 4 — shows the flow cytometry gating scheme applied to tumor samples [file crc-24-0434_supplemental_figure_4_suppsf4.pdf]

Supplemental Figure 4

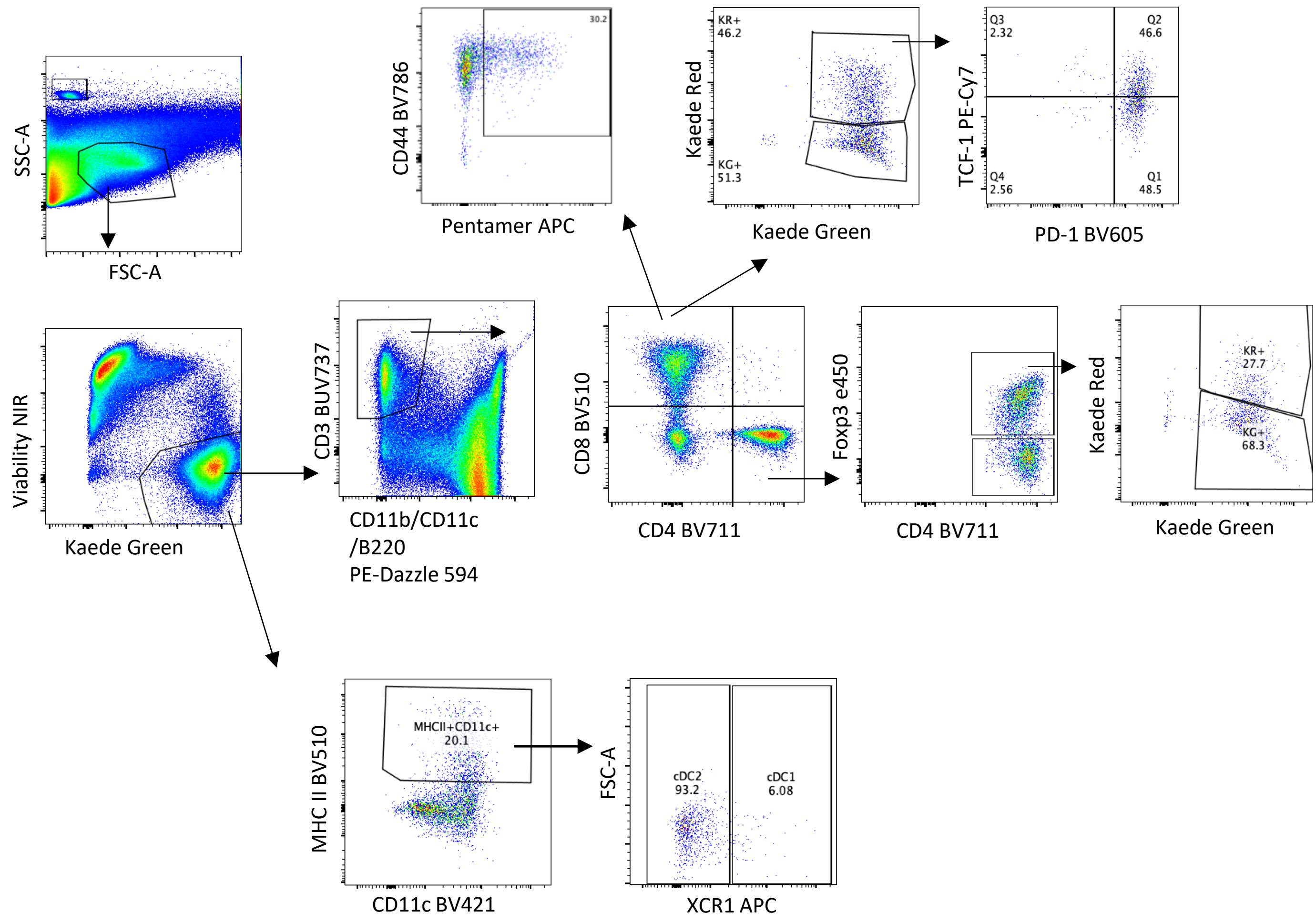

Supplemental Figure 4: Flow cytometric analysis of immune cells in tumors. The gating scheme applied to tumor samples is shown.
